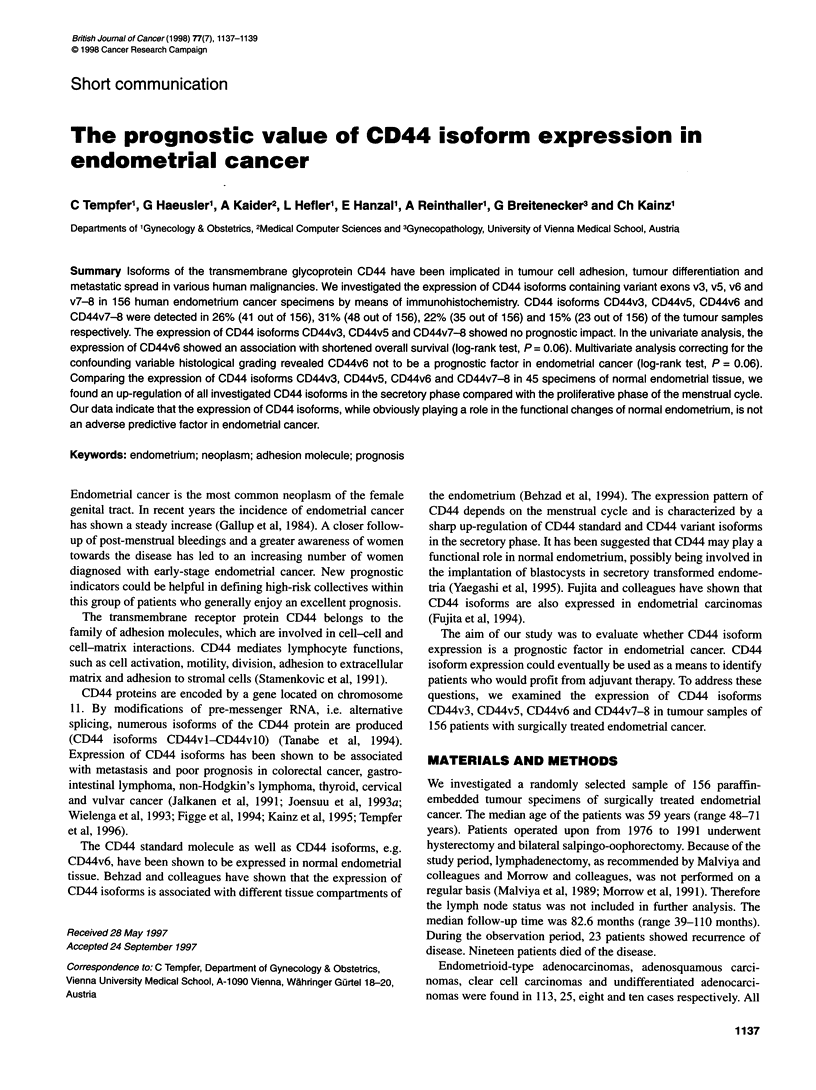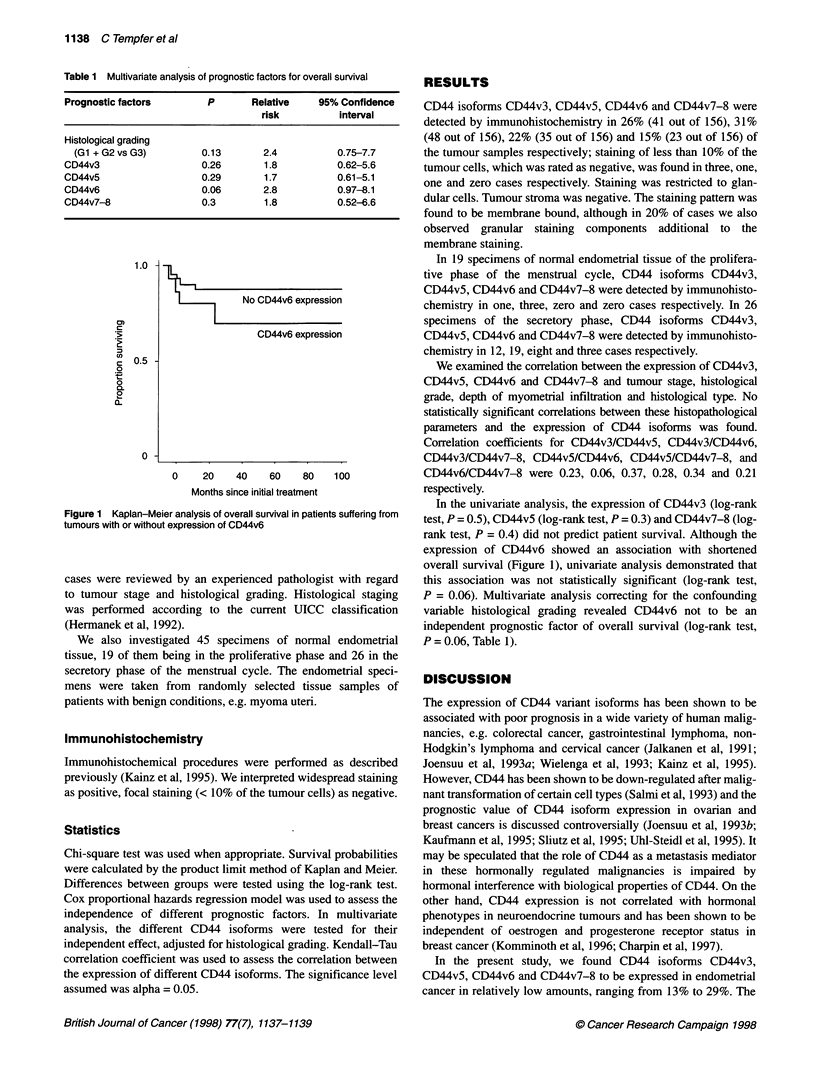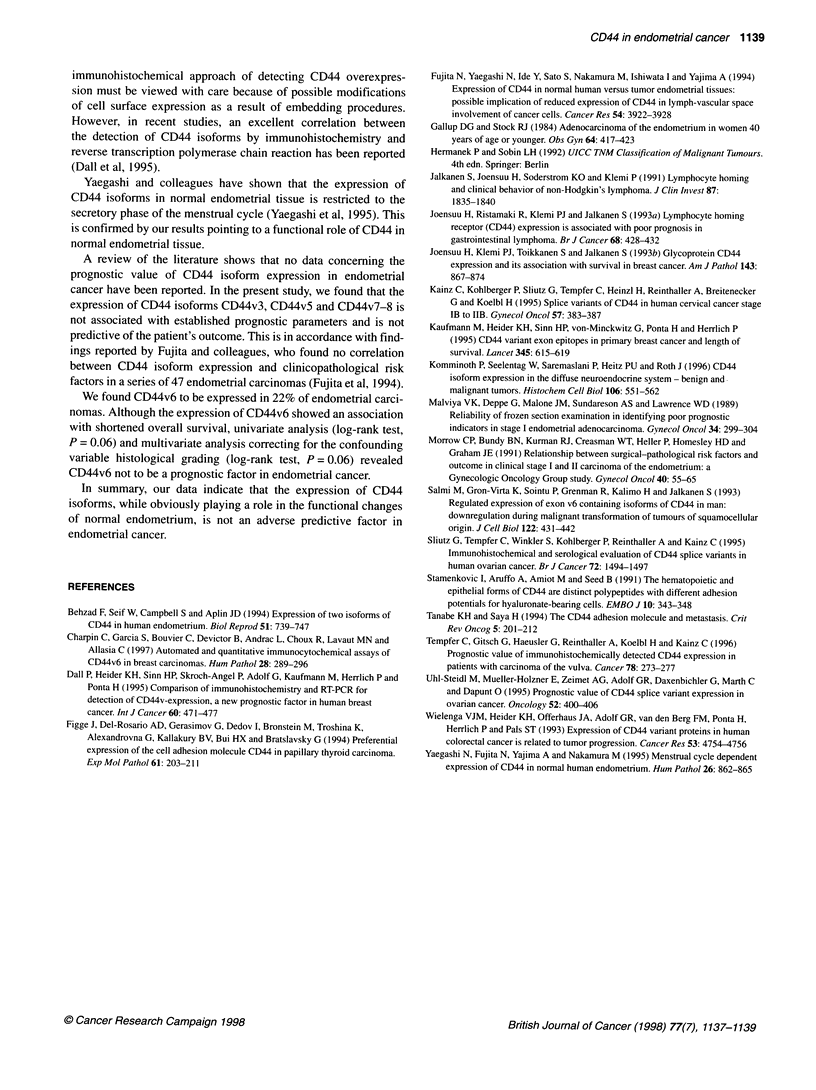# The prognostic value of CD44 isoform expression in endometrial cancer.

**DOI:** 10.1038/bjc.1998.188

**Published:** 1998-04

**Authors:** C. Tempfer, G. Haeusler, A. Kaider, L. Hefler, E. Hanzal, A. Reinthaller, G. Breitenecker, C. Kainz

**Affiliations:** Department of Gynecology & Obstetrics, University of Vienna Medical School, Austria.

## Abstract

Isoforms of the transmembrane glycoprotein CD44 have been implicated in tumour cell adhesion, tumour differentiation and metastatic spread in various human malignancies. We investigated the expression of CD44 isoforms containing variant exons v3, v5, v6 and v7-8 in 156 human endometrium cancer specimens by means of immunohistochemistry. CD44 isoforms CD44v3, CD44v5, CD44v6 and CD44v7-8 were detected in 26% (41 out of 156), 31% (48 out of 156), 22% (35 out of 156) and 15% (23 out of 156) of the tumour samples respectively. The expression of CD44 isoforms CD44v3, CD44v5 and CD44v7-8 showed no prognostic impact. In the univariate analysis, the expression of CD44v6 showed an association with shortened overall survival (log-rank test, P = 0.06). Multivariate analysis correcting for the confounding variable histological grading revealed CD44v6 not to be a prognostic factor in endometrial cancer (log-rank test, P = 0.06). Comparing the expression of CD44 isoforms CD44v3, CD44v5, CD44v6 and CD44v7-8 in 45 specimens of normal endometrial tissue, we found an up-regulation of all investigated CD44 isoforms in the secretory phase compared with the proliferative phase of the menstrual cycle. Our data indicate that the expression of CD44 isoforms, while obviously playing a role in the functional changes of normal endometrium, is not an adverse predictive factor in endometrial cancer.


British Journal of Cancer (1998) 77(7), 1137–1139
© 1998 Cancer Research Campaign

Short communication

### The prognostic value of CD44 isoform expression in endometrial cancer

**C Tempfer[1], G Haeusler[1], A Kaider[2], L Hefler[1], E Hanzal[1], A Reinthaller[1], G Breitenecker[3] and Ch Kainz[1]**

Departments of [1]Gynecology & Obstetrics, [2]Medical Computer Sciences and [3]Gynecopathology, University of Vienna Medical School, Austria

**Summary** Isoforms of the transmembrane glycoprotein CD44 have been implicated in tumour cell adhesion, tumour differentiation and metastatic spread in various human malignancies. We investigated the expression of CD44 isoforms containing variant exons v3, v5, v6 and v7–8 in 156 human endometrium cancer specimens by means of immunohistochemistry. CD44 isoforms CD44v3, CD44v5, CD44v6 and CD44v7–8 were detected in 26% (41 out of 156), 31% (48 out of 156), 22% (35 out of 156) and 15% (23 out of 156) of the tumour samples respectively. The expression of CD44 isoforms CD44v3, CD44v5 and CD44v7–8 showed no prognostic impact. In the univariate analysis, the expression of CD44v6 showed an association with shortened overall survival (log-rank test, $P = 0.06$). Multivariate analysis correcting for the confounding variable histological grading revealed CD44v6 not to be a prognostic factor in endometrial cancer (log-rank test, $P = 0.06$). Comparing the expression of CD44 isoforms CD44v3, CD44v5, CD44v6 and CD44v7–8 in 45 specimens of normal endometrial tissue, we found an up-regulation of all investigated CD44 isoforms in the secretory phase compared with the proliferative phase of the menstrual cycle. Our data indicate that the expression of CD44 isoforms, while obviously playing a role in the functional changes of normal endometrium, is not an adverse predictive factor in endometrial cancer.

**Keywords:** endometrium; neoplasm; adhesion molecule; prognosis

Endometrial cancer is the most common neoplasm of the female genital tract. In recent years the incidence of endometrial cancer has shown a steady increase (Gallup et al, 1984). A closer follow-up of post-menstrual bleedings and a greater awareness of women towards the disease has led to an increasing number of women diagnosed with early-stage endometrial cancer. New prognostic indicators could be helpful in defining high-risk collectives within this group of patients who generally enjoy an excellent prognosis.

The transmembrane receptor protein CD44 belongs to the family of adhesion molecules, which are involved in cell–cell and cell–matrix interactions. CD44 mediates lymphocyte functions, such as cell activation, motility, division, adhesion to extracellular matrix and adhesion to stromal cells (Stamenkovic et al, 1991).

CD44 proteins are encoded by a gene located on chromosome 11. By modifications of pre-messenger RNA, i.e. alternative splicing, numerous isoforms of the CD44 protein are produced (CD44 isoforms CD44v1–CD44v10) (Tanabe et al, 1994). Expression of CD44 isoforms has been shown to be associated with metastasis and poor prognosis in colorectal cancer, gastro-intestinal lymphoma, non-Hodgkin's lymphoma, thyroid, cervical and vulvar cancer (Jalkanen et al, 1991; Joensuu et al, 1993*a*; Wielenga et al, 1993; Figge et al, 1994; Kainz et al, 1995; Tempfer et al, 1996).

The CD44 standard molecule as well as CD44 isoforms, e.g. CD44v6, have been shown to be expressed in normal endometrial tissue. Behzad and colleagues have shown that the expression of CD44 isoforms is associated with different tissue compartments of the endometrium (Behzad et al, 1994). The expression pattern of CD44 depends on the menstrual cycle and is characterized by a sharp up-regulation of CD44 standard and CD44 variant isoforms in the secretory phase. It has been suggested that CD44 may play a functional role in normal endometrium, possibly being involved in the implantation of blastocysts in secretory transformed endometria (Yaegashi et al, 1995). Fujita and colleagues have shown that CD44 isoforms are also expressed in endometrial carcinomas (Fujita et al, 1994).

The aim of our study was to evaluate whether CD44 isoform expression is a prognostic factor in endometrial cancer. CD44 isoform expression could eventually be used as a means to identify patients who would profit from adjuvant therapy. To address these questions, we examined the expression of CD44 isoforms CD44v3, CD44v5, CD44v6 and CD44v7–8 in tumour samples of 156 patients with surgically treated endometrial cancer.

### MATERIALS AND METHODS

We investigated a randomly selected sample of 156 paraffin-embedded tumour specimens of surgically treated endometrial cancer. The median age of the patients was 59 years (range 48–71 years). Patients operated upon from 1976 to 1991 underwent hysterectomy and bilateral salpingo-oophorectomy. Because of the study period, lymphadenectomy, as recommended by Malviya and colleagues and Morrow and colleagues, was not performed on a regular basis (Malviya et al, 1989; Morrow et al, 1991). Therefore the lymph node status was not included in further analysis. The median follow-up time was 82.6 months (range 39–110 months). During the observation period, 23 patients showed recurrence of disease. Nineteen patients died of the disease.

Endometrioid-type adenocarcinomas, adenosquamous carcinomas, clear cell carcinomas and undifferentiated adenocarcinomas were found in 113, 25, eight and ten cases respectively. All

*Received 28 May 1997*
*Accepted 24 September 1997*

*Correspondence to:* C Tempfer, Department of Gynecology & Obstetrics, Vienna University Medical School, A-1090 Vienna, Währinger Gürtel 18–20, Austria

**Table 1** Multivariate analysis of prognostic factors for overall survival

| Prognostic factors | P | Relative risk | 95% Confidence interval |
|---|---|---|---|
| Histological grading | | | |
| (G1 + G2 vs G3) | 0.13 | 2.4 | 0.75–7.7 |
| CD44v3 | 0.26 | 1.8 | 0.62–5.6 |
| CD44v5 | 0.29 | 1.7 | 0.61–5.1 |
| CD44v6 | 0.06 | 2.8 | 0.97–8.1 |
| CD44v7–8 | 0.3 | 1.8 | 0.52–6.6 |

**Figure 1** Kaplan–Meier analysis of overall survival in patients suffering from tumours with or without expression of CD44v6

cases were reviewed by an experienced pathologist with regard to tumour stage and histological grading. Histological staging was performed according to the current UICC classification (Hermanek et al, 1992).

We also investigated 45 specimens of normal endometrial tissue, 19 of them being in the proliferative phase and 26 in the secretory phase of the menstrual cycle. The endometrial specimens were taken from randomly selected tissue samples of patients with benign conditions, e.g. myoma uteri.

### Immunohistochemistry

Immunohistochemical procedures were performed as described previously (Kainz et al, 1995). We interpreted widespread staining as positive, focal staining (< 10% of the tumour cells) as negative.

### Statistics

Chi-square test was used when appropriate. Survival probabilities were calculated by the product limit method of Kaplan and Meier. Differences between groups were tested using the log-rank test. Cox proportional hazards regression model was used to assess the independence of different prognostic factors. In multivariate analysis, the different CD44 isoforms were tested for their independent effect, adjusted for histological grading. Kendall–Tau correlation coefficient was used to assess the correlation between the expression of different CD44 isoforms. The significance level assumed was alpha = 0.05.

### RESULTS

CD44 isoforms CD44v3, CD44v5, CD44v6 and CD44v7–8 were detected by immunohistochemistry in 26% (41 out of 156), 31% (48 out of 156), 22% (35 out of 156) and 15% (23 out of 156) of the tumour samples respectively; staining of less than 10% of the tumour cells, which was rated as negative, was found in three, one, one and zero cases respectively. Staining was restricted to glandular cells. Tumour stroma was negative. The staining pattern was found to be membrane bound, although in 20% of cases we also observed granular staining components additional to the membrane staining.

In 19 specimens of normal endometrial tissue of the proliferative phase of the menstrual cycle, CD44 isoforms CD44v3, CD44v5, CD44v6 and CD44v7–8 were detected by immunohistochemistry in one, three, zero and zero cases respectively. In 26 specimens of the secretory phase, CD44 isoforms CD44v3, CD44v5, CD44v6 and CD44v7–8 were detected by immunohistochemistry in 12, 19, eight and three cases respectively.

We examined the correlation between the expression of CD44v3, CD44v5, CD44v6 and CD44v7–8 and tumour stage, histological grade, depth of myometrial infiltration and histological type. No statistically significant correlations between these histopathological parameters and the expression of CD44 isoforms was found. Correlation coefficients for CD44v3/CD44v5, CD44v3/CD44v6, CD44v3/CD44v7–8, CD44v5/CD44v6, CD44v5/CD44v7–8, and CD44v6/CD44v7–8 were 0.23, 0.06, 0.37, 0.28, 0.34 and 0.21 respectively.

In the univariate analysis, the expression of CD44v3 (log-rank test, $P = 0.5$), CD44v5 (log-rank test, $P = 0.3$) and CD44v7–8 (log-rank test, $P = 0.4$) did not predict patient survival. Although the expression of CD44v6 showed an association with shortened overall survival (Figure 1), univariate analysis demonstrated that this association was not statistically significant (log-rank test, $P = 0.06$). Multivariate analysis correcting for the confounding variable histological grading revealed CD44v6 not to be an independent prognostic factor of overall survival (log-rank test, $P = 0.06$, Table 1).

### DISCUSSION

The expression of CD44 variant isoforms has been shown to be associated with poor prognosis in a wide variety of human malignancies, e.g. colorectal cancer, gastrointestinal lymphoma, non-Hodgkin's lymphoma and cervical cancer (Jalkanen et al, 1991; Joensuu et al, 1993*a*; Wielenga et al, 1993; Kainz et al, 1995). However, CD44 has been shown to be down-regulated after malignant transformation of certain cell types (Salmi et al, 1993) and the prognostic value of CD44 isoform expression in ovarian and breast cancers is discussed controversially (Joensuu et al, 1993*b*; Kaufmann et al, 1995; Sliutz et al, 1995; Uhl-Steidl et al, 1995). It may be speculated that the role of CD44 as a metastasis mediator in these hormonally regulated malignancies is impaired by hormonal interference with biological properties of CD44. On the other hand, CD44 expression is not correlated with hormonal phenotypes in neuroendocrine tumours and has been shown to be independent of oestrogen and progesterone receptor status in breast cancer (Komminoth et al, 1996; Charpin et al, 1997).

In the present study, we found CD44 isoforms CD44v3, CD44v5, CD44v6 and CD44v7–8 to be expressed in endometrial cancer in relatively low amounts, ranging from 13% to 29%. The

© Cancer Research Campaign 1998

immunohistochemical approach of detecting CD44 overexpression must be viewed with care because of possible modifications of cell surface expression as a result of embedding procedures. However, in recent studies, an excellent correlation between the detection of CD44 isoforms by immunohistochemistry and reverse transcription polymerase chain reaction has been reported (Dall et al, 1995).

Yaegashi and colleagues have shown that the expression of CD44 isoforms in normal endometrial tissue is restricted to the secretory phase of the menstrual cycle (Yaegashi et al, 1995). This is confirmed by our results pointing to a functional role of CD44 in normal endometrial tissue.

A review of the literature shows that no data concerning the prognostic value of CD44 isoform expression in endometrial cancer have been reported. In the present study, we found that the expression of CD44 isoforms CD44v3, CD44v5 and CD44v7–8 is not associated with established prognostic parameters and is not predictive of the patient's outcome. This is in accordance with findings reported by Fujita and colleagues, who found no correlation between CD44 isoform expression and clinicopathological risk factors in a series of 47 endometrial carcinomas (Fujita et al, 1994).

We found CD44v6 to be expressed in 22% of endometrial carcinomas. Although the expression of CD44v6 showed an association with shortened overall survival, univariate analysis (log-rank test, $P = 0.06$) and multivariate analysis correcting for the confounding variable histological grading (log-rank test, $P = 0.06$) revealed CD44v6 not to be a prognostic factor in endometrial cancer.

In summary, our data indicate that the expression of CD44 isoforms, while obviously playing a role in the functional changes of normal endometrium, is not an adverse predictive factor in endometrial cancer.

### REFERENCES

Behzad F, Seif W, Campbell S and Aplin JD (1994) Expression of two isoforms of CD44 in human endometrium. *Biol Reprod* **51**: 739–747

Charpin C, Garcia S, Bouvier C, Devictor B, Andrac L, Choux R, Lavaut MN and Allasia C (1997) Automated and quantitative immunocytochemical assays of CD44v6 in breast carcinomas. *Hum Pathol* **28**: 289–296

Dall P, Heider KH, Sinn HP, Skroch-Angel P, Adolf G, Kaufmann M, Herrlich P and Ponta H (1995) Comparison of immunohistochemistry and RT-PCR for detection of CD44v-expression, a new prognostic factor in human breast cancer. *Int J Cancer* **60**: 471–477

Figge J, Del-Rosario AD, Gerasimov G, Dedov I, Bronstein M, Troshina K, Alexandrovna G, Kallakury BV, Bui HX and Bratslavsky G (1994) Preferential expression of the cell adhesion molecule CD44 in papillary thyroid carcinoma. *Exp Mol Pathol* **61**: 203–211

Fujita N, Yaegashi N, Ide Y, Sato S, Nakamura M, Ishiwata I and Yajima A (1994) Expression of CD44 in normal human versus tumor endometrial tissues: possible implication of reduced expression of CD44 in lymph-vascular space involvement of cancer cells. *Cancer Res* **54**: 3922–3928

Gallup DG and Stock RJ (1984) Adenocarcinoma of the endometrium in women 40 years of age or younger. *Obs Gyn* **64**: 417–423

Hermanek P and Sobin LH (1992) *UICC TNM Classification of Malignant Tumours*. 4th edn. Springer: Berlin

Jalkanen S, Joensuu H, Soderstrom KO and Klemi P (1991) Lymphocyte homing and clinical behavior of non-Hodgkin's lymphoma. *J Clin Invest* **87**: 1835–1840

Joensuu H, Ristamaki R, Klemi PJ and Jalkanen S (1993*a*) Lymphocyte homing receptor (CD44) expression is associated with poor prognosis in gastrointestinal lymphoma. *Br J Cancer* **68**: 428–432

Joensuu H, Klemi PJ, Toikkanen S and Jalkanen S (1993*b*) Glycoprotein CD44 expression and its association with survival in breast cancer. *Am J Pathol* **143**: 867–874

Kainz C, Kohlberger P, Sliutz G, Tempfer C, Heinzl H, Reinthaller A, Breitenecker G and Koelbl H (1995) Splice variants of CD44 in human cervical cancer stage IB to IIB. *Gynecol Oncol* **57**: 383–387

Kaufmann M, Heider KH, Sinn HP, von-Minckwitz G, Ponta H and Herrlich P (1995) CD44 variant exon epitopes in primary breast cancer and length of survival. *Lancet* **345**: 615–619

Komminoth P, Seelentag W, Saremaslani P, Heitz PU and Roth J (1996) CD44 isoform expression in the diffuse neuroendocrine system – benign and malignant tumors. *Histochem Cell Biol* **106**: 551–562

Malviya VK, Deppe G, Malone JM, Sundareson AS and Lawrence WD (1989) Reliability of frozen section examination in identifying poor prognostic indicators in stage I endometrial adenocarcinoma. *Gynecol Oncol* **34**: 299–304

Morrow CP, Bundy BN, Kurman RJ, Creasman WT, Heller P, Homesley HD and Graham JE (1991) Relationship between surgical–pathological risk factors and outcome in clinical stage I and II carcinoma of the endometrium: a Gynecologic Oncology Group study. *Gynecol Oncol* **40**: 55–65

Salmi M, Gron-Virta K, Sointu P, Grenman R, Kalimo H and Jalkanen S (1993) Regulated expression of exon v6 containing isoforms of CD44 in man: downregulation during malignant transformation of tumours of squamocellular origin. *J Cell Biol* **122**: 431–442

Sliutz G, Tempfer C, Winkler S, Kohlberger P, Reinthaller A and Kainz C (1995) Immunohistochemical and serological evaluation of CD44 splice variants in human ovarian cancer. *Br J Cancer* **72**: 1494–1497

Stamenkovic I, Aruffo A, Amiot M and Seed B (1991) The hematopoietic and epithelial forms of CD44 are distinct polypeptides with different adhesion potentials for hyaluronate-bearing cells. *EMBO J* **10**: 343–348

Tanabe KH and Saya H (1994) The CD44 adhesion molecule and metastasis. *Crit Rev Oncog* **5**: 201–212

Tempfer C, Gitsch G, Haeusler G, Reinthaller A, Koelbl H and Kainz C (1996) Prognostic value of immunohistochemically detected CD44 expression in patients with carcinoma of the vulva. *Cancer* **78**: 273–277

Uhl-Steidl M, Mueller-Holzner E, Zeimet AG, Adolf GR, Daxenbichler G, Marth C and Dapunt O (1995) Prognostic value of CD44 splice variant expression in ovarian cancer. *Oncology* **52**: 400–406

Wielenga VJM, Heider KH, Offerhaus JA, Adolf GR, van den Berg FM, Ponta H, Herrlich P and Pals ST (1993) Expression of CD44 variant proteins in human colorectal cancer is related to tumor progression. *Cancer Res* **53**: 4754–4756

Yaegashi N, Fujita N, Yajima A and Nakamura M (1995) Menstrual cycle dependent expression of CD44 in normal human endometrium. *Hum Pathol* **26**: 862–865

© Cancer Research Campaign 1998